# Supplementary material for: Real-World Management of High Stool Output in Patients with Short Bowel Syndrome: An International Multicenter Survey
Source: Nutrients. 2023 Jun 16;15(12):2763. doi: 10.3390/nu15122763 (PMC10305686; doi:10.3390/nu15122763)
Supplement: Supplementary file 1 [file nutrients-15-02763-s001.zip › nutrients-2418858-supplementary.pdf]

SBS Management Survey 1.0

Dr. Leah Gramlich & Dr. Narisorn Lakananurak
Division of Gastroenterology
University of Alberta

Welcome to the Real World Management of Short Bowel Syndrome Survey.

Intestinal failure (IF) is rare condition defined as "the reduction of gut function below the minimum necessary for the absorption of macronutrients and/or water and electrolytes, such that parental/intravenous supplementation (Parental Support, PS) is required to maintain health and/or growth" (Pironi et al. Clin Nutrition 2016, 2018, 2020).

IF can be classified as Type I acute intestinal failure (AIF) Type II prolonged AIF and Type III chronic intestinal failure (CIF) and it may be due to one or more of five major pathophysiological mechanisms. Evidence Based Guidelines and management concepts for IF in adults, have been outlined endorsed by experts in the European Society for Clinical Nutrition and Metabolism (ESPEN) (Pironi et al 2018).

Short bowel syndrome (SBS) is the most frequent pathophysiological mechanism of CIF seen in about two thirds of patients. In these patients, the main objective of SBS CIF treatment is to optimize gastrointestinal function using an integrated approach to medical and nutritional management and to provide optimal parenteral support. The Evidence-based guidelines (EBG) have facilitated a standard approach across the world. However, despite the advent of EBG's there is a variable approach in practice to IF and a lack of specific guidance from experts who have real world experience in managing the high stool output associated with SBS IF.

The aim of this survey is to identify and illuminate real-world practice in expert IF management centers. The goal is to develop specific guidance to improve management in SBS CIF in adult patients. In addition to questions about your IF program, questions addressing management of stool output are grouped into 4 areas: Diet, Fluids, Medications, GLP-2 Therapy,

As a site investigator for EASE trials, your IF/HPN team has been identified as a center of excellence for the management of SBS IF. Your input into this work is anticipated to have implications for both acute and chronic IF management and the adult patients who live with this condition.

Thank you for participating in our survey. Your feedback is important.

Please enter as multi-disciplinary team. One questionnaire per team per site.

SBS Management Survey 1.0

Program characteristics

* 1. What are the disciplines in your team?

- [ ] Dietitian
- [ ] Nurse
- [ ] Nurse Practitioner
- [ ] Pharmacist
- [ ] Gastroenterologist
- [ ] Endocrinologist
- [ ] Surgeon
- [ ] Pediatrician
- [ ] Internist (Internal medicine)
- [ ] Physician assistant
- [ ] Psychologist
- [ ] Social worker
- [ ] Financial Counselor
- [ ] Research Coordinator
- [ ] Other (please specify)

* 2. In what country is your medical center?

Country

* 3. Do you have dedicated IF beds supported by specialized knowledgeable team in your hospital?

- [ ] Yes
- [ ] No
- [ ] Other (please specify)

* 4. What is the full-time equivalent (FTE) of each discipline that makes up your team?

Please provide numbers of each discipline, e.g. 1.0 FTE works full time , 0.5 FTE works half time in a discipline, 2 nurses at 1.0 FTE would = 2.0 FTE

Dietitians	
Nurses	
Nurse Practioner	
Pharmacists	
Gastroenterologists	
Endocrinologists	
Surgeons	
Pediatricians	
Internist (Internal medicine)	
Physician assistant	
Psychologists	
Social workers	
Financial Counselor	
Research Coordinator	
Others	

* 5. How many CIF patients [see definition on page 1] in total are in your program?

Enter a number

* 6. What is the estimated number of patients whose main diagnosis is in the following pathophysiological classifications of IF? (per ESPEN guidelines)

Specify the proportion to the closest tenth percent

	None 0 %	10 %	20 %	30 %	40 %	50 %	60 %	70 %	80 %	90 %	All 100 %
Short bowel	○	○	○	○	○	○	○	○	○	○	○
Intestinal fistula	○	○	○	○	○	○	○	○	○	○	○
Intestinal dysmotility	○	○	○	○	○	○	○	○	○	○	○
Mechanical obstruction	○	○	○	○	○	○	○	○	○	○	○
Extensive small bowel mucosal disease	○	○	○	○	○	○	○	○	○	○	○
Other	○	○	○	○	○	○	○	○	○	○	○

Other causes (please specify, one line per cause)

* 7. What is the estimated proportion of patients in each pathophysiological classification of SBS IF?

Specify the proportion to the closest tenth percent

	None 0 %	10 %	20 %	30 %	40 %	50 %	60 %	70 %	80 %	90 %	All 100 %
Mesenteric ischemia	○	○	○	○	○	○	○	○	○	○	○
Inflammatory bowel diseases	○	○	○	○	○	○	○	○	○	○	○
Radiation enteritis	○	○	○	○	○	○	○	○	○	○	○
Surgical complication	○	○	○	○	○	○	○	○	○	○	○
Volvulus and hernia	○	○	○	○	○	○	○	○	○	○	○
Cancer	○	○	○	○	○	○	○	○	○	○	○
Other	○	○	○	○	○	○	○	○	○	○	○

Other causes (please specify, one line per cause)

* 8. What proportion of your patients are anticipated to be shorter term (HPN < 1 year) versus lifelong?

Specify to the closest tenth percentage

	Never 0 %	10 %	20 %	30 %	40 %	50 %	60 %	70 %	80 %	90 %	Always 100 %
Shorter term patients (HPN < 1 year)	○	○	○	○	○	○	○	○	○	○	○

SBS Management Survey 1.0

Better Management of high stool output: Diet

* 9. How often do you recommend the following diets to patients with colon in-continuity with high stool output?

Specify the proportion to the closest tenth percent

	Never 0%	10%	20%	30%	40%	50%	60%	70%	80%	90%	Always 100%
Low simple sugar diet	○	○	○	○	○	○	○	○	○	○	○
Low fat diet	○	○	○	○	○	○	○	○	○	○	○
Addition of soluble fiber	○	○	○	○	○	○	○	○	○	○	○
Separation fluid and solid food	○	○	○	○	○	○	○	○	○	○	○
High Sodium diet	○	○	○	○	○	○	○	○	○	○	○
Reduce oxalate	○	○	○	○	○	○	○	○	○	○	○
Oral nutritional Supplements	○	○	○	○	○	○	○	○	○	○	○
Other diets	○	○	○	○	○	○	○	○	○	○	○

Other diets (please specify)

* 10. How often do you recommend the following diets to patients without colon in-continuity with high stool output?

Specify the proportion to the closest tenth percent

	Never 0%	10%	20%	30%	40%	50%	60%	70%	80%	90%	Always 100%
Low simple sugar diet	○	○	○	○	○	○	○	○	○	○	○
Low fat diet	○	○	○	○	○	○	○	○	○	○	○
Addition of soluble fiber	○	○	○	○	○	○	○	○	○	○	○
Separation fluid and solid food	○	○	○	○	○	○	○	○	○	○	○
High Sodium diet	○	○	○	○	○	○	○	○	○	○	○
Reduce oxalate	○	○	○	○	○	○	○	○	○	○	○
Oral nutritional Supplements	○	○	○	○	○	○	○	○	○	○	○
Other diets	○	○	○	○	○	○	○	○	○	○	○

Other diets (please specify)

Better Management of high stool output: Fluids

* 11. Do you recommend to **restrict** the following types of oral fluid in patients with high stool output?

Select all that apply

- [ ] Water
- [ ] Tea
- [ ] Coffee (regular)
- [ ] Coffee (decaf)
- [ ] Fruit juice
- [ ] Sweetened Beverages (lemonade, sweat tea, fruit punch, soft-drinks/Coke/Pepsi/Sprite/Ginger Ale)
- [ ] Alcohol
- [ ] Other oral fluids (please specify)

- [ ] No, we provide general guidance on fluid management (e.g. osmolarity and solute load)

* 12. Do you recommend the following types of ORS (oral rehydration solution)?

Select all that apply

- [ ] Homemade ORS
- [ ] Commercial products (e.g. WHO ORS, Pedialyte)
- [ ] Sport drinks (e.g. G2)
- [ ] Other ORS (please specify)

* 13. What proportion of patients experience the following as barriers to use ORS as listed in Question 10?

Select all that apply

	None 0%	10%	20%	30%	40%	50%	60%	70%	80%	90%	All 100%
Taste fatigue											
Cost											
Accessibility											
Gastrointestinal symptoms (i.e. nausea, vomiting, abdominal pain)											
Inadequate tools and/or education for ORS											
Other											

Other (please specify)

* 14. What proportion of patients experience the following as enablers to use ORS as listed in Question 10?

Specify to closest percent

	None 0%	10%	20%	30%	40%	50%	60%	70%	80%	90%	All 100%
Good taste											
Affordable price											
Reimbursement											
Availability											
Treatment of gastrointestinal symptoms (nausea, vomiting, abdominal pain)											
Decreased stool output											
Improved sense of hydration											
Other											

Other (please specify)

* 15. Which IV solutions do you use for simple rehydration of SBS IF patients

Specify the proportion to the closest tenth percent

	Never 0%	10%	20%	30%	40%	50%	60%	70%	80%	90%	Always 100%
Normal saline	○	○	○	○	○	○	○	○	○	○	○
½ Normal saline	○	○	○	○	○	○	○	○	○	○	○
Dextrose water	○	○	○	○	○	○	○	○	○	○	○
Dextrose / glucose 5%	○	○	○	○	○	○	○	○	○	○	○
Ringer lactate solution	○	○	○	○	○	○	○	○	○	○	○
Compounded solution	○	○	○	○	○	○	○	○	○	○	○
Other IV hydration	○	○	○	○	○	○	○	○	○	○	○

Better Management of high stool output: Medications

* 16. Which of the following guides your choice of anti-diarrhoeal medication?

Specify the proportion to the closest tenth percent

	None 0 %	10 %	20 %	30 %	40 %	50 %	60 %	70 %	80 %	90 %	All 100 %
Remnant anatomy	○	○	○	○	○	○	○	○	○	○	○
Type of ostomy	○	○	○	○	○	○	○	○	○	○	○
Stool volume	○	○	○	○	○	○	○	○	○	○	○
Urine output	○	○	○	○	○	○	○	○	○	○	○
Presence of ileo-caecal valve	○	○	○	○	○	○	○	○	○	○	○
Underlying disease	○	○	○	○	○	○	○	○	○	○	○
Other	○	○	○	○	○	○	○	○	○	○	○

Other drug (please specify)

* 17. Do you within your practice use the following antimotility drugs?

Specify the proportion to the closest tenth percent

	None 0 %	10 %	20 %	30 %	40 %	50 %	60 %	70 %	80 %	90 %	All 100 %
Loperamide	○	○	○	○	○	○	○	○	○	○	○
Diphenoxylate and atropine (lomotil)	○	○	○	○	○	○	○	○	○	○	○
Codeine	○	○	○	○	○	○	○	○	○	○	○
Tincture opium	○	○	○	○	○	○	○	○	○	○	○
Other	○	○	○	○	○	○	○	○	○	○	○

Other drug (please specify)

* 18. What daily dose do you <u>usually start</u> with for each antimotility medication?

Enter total daily dose for each medication

<u>Loperamide</u>
Total daily dose (mg) [ ]

<u>Diphenoxylate and atropine</u> *(1 Lomotil = 2.5mg diphenoxylate and 0.025 mg atropine sulphate)*
Total daily dose of diphenoxylate (mg) [ ]

<u>Codeine</u>
Total daily dose (mg) [ ]

<u>Tincture opium</u>
Total daily dose (mg) [ ]

<u>Other medication</u>
Medication name and Total daily dose [ ]

* 19. In your practice, what is the indication to increase the dose of antimotility medications?

Select all that apply

- [ ] Increased stool output
- [ ] Increased stool frequency
- [ ] Loosening stool
- [ ] Thirst and dehydration
- [ ] Increased BUN/Cr (blood urea nitrogen / creatinine ratio)
- [ ] Insufficient effect of current dose
- [ ] Other (please specify)

[ ]

* 20. What is the highest daily dose and frequency you usually use for each antimotility medication?

Enter a highest dose and frequency per day for each medication

Loperamide
Total daily dose (mg)

Diphenoxylate and atropine *(1 Lomotil = 2.5 mg diphenoxylate and 0.025 mg atropine sulphate)*
Total daily dose of diphenoxylate (mg)

Codeine
Total daily dose (mg)

Tincture opium
Total daily dose (mg)

Other medication
Medication name and Total daily dose

* 21. What are the first, second and third choice of antimotility medications for the average SBS IF patient?

	Loperamide	Diphenoxylate and atropine (lomotil)	Codeine	Tincture opium	Other medication
1st choice antimotility medication	○	○	○	○	○
2nd choice antimotility medication	○	○	○	○	○
3rd choice antimotility medication	○	○	○	○	○

* 22. Do you change your strategy for patients above 75 years?

- Yes
- No

* 23. For an average patient older than 75 years of age, what are the first, second and third choice antimotility medications?

	Loperamide	Diphenoxylate and atropine (lomotil)	Codeine	Tincture opium	Other medication
1st choice antimotility medication	○	○	○	○	○
2nd choice antimotility medication	○	○	○	○	○
3rd choice antimotility medication	○	○	○	○	○

* 24. What proportion of patients receive more than one antimotility drug?

Specify to the closest tenth percentage

	Never 0 %	10 %	20 %	30 %	40 %	50 %	60 %	70 %	80 %	90 %	Always 100 %
SBS IF patients receiving >1 antimotility medication	○	○	○	○	○	○	○	○	○	○	○

* 25. Is your choice of antimotility drugs influenced by residual intestinal anatomy?

○ No

○ Yes

If Yes, how?

Better Management of high stool output: Medications

* 26. How within your practice do you use the following antisecretory drugs?

Specify to the closest tenth percent

	Never 0%	10%	20%	30%	40%	50%	60%	70%	80%	90%	Always 100%
Proton Pump Inhibitor (capsule, tablet)	◯	◯	◯	◯	◯	◯	◯	◯	◯	◯	◯
Proton Pump Inhibitor (Multiple-Unit Pellet System (MUPS) given as tablets, dissolves into subunits across stomach and small intestine)	◯	◯	◯	◯	◯	◯	◯	◯	◯	◯	◯
H2 Receptor Antagonist (oral)	◯	◯	◯	◯	◯	◯	◯	◯	◯	◯	◯
H2 Receptor Antagonist (add to PN)	◯	◯	◯	◯	◯	◯	◯	◯	◯	◯	◯
Somatostatin analogue (e.g. Octreotide)	◯	◯	◯	◯	◯	◯	◯	◯	◯	◯	◯
Other antisecretory drug	◯	◯	◯	◯	◯	◯	◯	◯	◯	◯	◯

* 27. What are the first, second and third line antisecretory medications?

	Proton Pump Inhibitor (capsule, tablet)	Proton Pump Inhibitor (MUPS)	H2 Receptor Antagonist (oral)	H2 Receptor Antagonist (add to PN)	Somatostatin analogue	Other medication
1st line antisecretory medication	◯	◯	◯	◯	◯	◯
2nd line antisecretory medication	◯	◯	◯	◯	◯	◯
3rd line antisecretory medication	◯	◯	◯	◯	◯	◯

* 28. What total daily dose do you usually start with for each antisecretory medication?

Enter a generic name and start total daily dose for each antisecretory medication

PPI
which PPI?

Total daily dose (mg)

H2 Receptor Antagonist (Oral)
Which H2RA?

Total daily dose (mg)

H2 Receptor Antagonist (add to PS)
Which H2RA?

Total daily dose (mg)

Somatostatin analogue
Total Daily dose (mg)

* 29. What highest daily dose do you prescribe for each antisecretory medication?

Enter a generic name and highest total daily dose for each antisecretory medication

PPI
which PPI?

Highest total daily dose (mg)

H2 Receptor Antagonist (Oral)
Which H2RA?

Highest total daily dose (mg)

H2 Receptor Antagonist (add to PS)
Which H2RA?

Highest total daily dose (mg)

Somatostatin analogue
Highest total Daily dose (mg)

* 30. Do you stop PPI after SBS IF diagnosis?

Select one answer

- ◯ After 6 months
- ◯ Never stop
- ◯ Other (please specify)

31. Do you stop Oral H2RA after SBS IF diagnosis?

Select one answer

- ◯ After 6 months
- ◯ Never stop
- ◯ Other (please specify)

* 32. Do you stop PS H2RA after SBS IF diagnosis?

Select one answer

- ◯ After 6 months
- ◯ Never stop
- ◯ Other (please specify)

* 33. How often in your practice do you trial the following medicines or supplements in SBS IF with colon in-continuity (CiC)?

Specify to the closest tenth percent

	Never 0%	10%	20%	30%	40%	50%	60%	70%	80%	90%	Always 100%
Fiber, e.g. psyllium fiber supplement	◯	◯	◯	◯	◯	◯	◯	◯	◯	◯	◯
Bile acid binders, e.g. questran	◯	◯	◯	◯	◯	◯	◯	◯	◯	◯	◯
Probiotics	◯	◯	◯	◯	◯	◯	◯	◯	◯	◯	◯
Pancreatic enzymes	◯	◯	◯	◯	◯	◯	◯	◯	◯	◯	◯

* 34. Within your practice, how often do you trial the following medicines or supplements in SBS IF without colon in-continuity?

Specify to the closest tenth percent

	Never 0%	10%	20%	30%	40%	50%	60%	70%	80%	90%	Always 100%
Fiber e.g. metamucil	◯	◯	◯	◯	◯	◯	◯	◯	◯	◯	◯
Bile acid binders e.g. questran	◯	◯	◯	◯	◯	◯	◯	◯	◯	◯	◯
Probiotics	◯	◯	◯	◯	◯	◯	◯	◯	◯	◯	◯
Pancreatic enzymes	◯	◯	◯	◯	◯	◯	◯	◯	◯	◯	◯
